# Innate immunity and training to subvert original antigenic sin by the humoral immune response

**DOI:** 10.7554/eLife.106654

**Published:** 2025-08-28

**Authors:** Faez Amokrane Nait Mohamed, Daniel Lingwood

**Affiliations:** 1 The Ragon Institute of Mass General Brigham, MIT and Harvard Boston United States; Radboud University Medical Center Netherlands; Indian Institute of Science Education and Research (IISER) India

**Keywords:** B cell, imprinting, repertoire

## Abstract

Originally defined in the context of influenza vaccines by Thomas Francis Jr. in the late 1950s, original antigenic sin (OAS) refers to the tendency of the immune system to preferentially recall B cell memory against primary antigen after secondary exposure to different but related antigen. This competes with the elicitation of *de novo* antibodies by lowering the frequency of antigen reception by the naïve B cell lymphocyte pool residing within secondary lymphoid organs. Consequently, OAS imposes a ‘primary addiction’ that modulates the target epitope specificity of the secondary antibody response and has wide-reaching consequences for vaccines that require seasonal updating, including influenza and SARS-CoV-2. Rationally designed vaccines that preferentially stimulate the production of *de novo* antibodies rather than those derived from recalled B cell memory are of central interest, particularly for universal vaccine formulations tasked with directing robust humoral immunity against these viruses which, due to their ongoing evolution, have ‘resisted’ conventional vaccine approaches. Largely absent from this discussion is an integrated evaluation of what Janeway famously called ‘the immunologists dirty secret’, that humoral immune reactions require stimulation by the innate immune system. In this perspective piece, we present a hypothesis that innate immune cells and trained immunity, a collective term for the epigenetic reprogramming that enhances responsiveness upon re-stimulation, provides a template for promoting *de novo* expansion of the naïve B cell repertoire over recallable memory. This natural control axis may inform the design of vaccines that seek to avoid primary addiction and OAS.

## General

**Figure 1.**
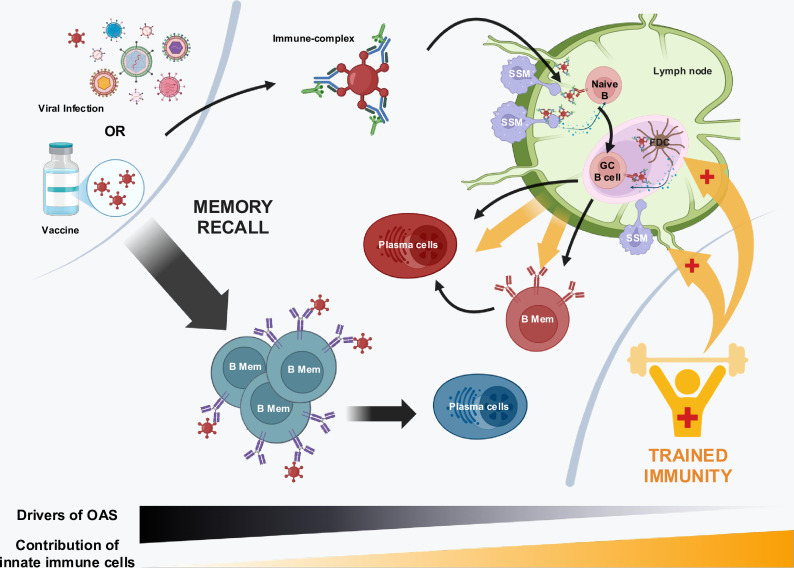
Selective innate immune support for expanding low-affinity B cells from the naïve B cell repertoire following immune challenge. Recall of higher affinity B cells does not receive this support. Following infection or vaccination, memory B cells are rapidly recalled, which can underscore original antigenic sin (OAS) or primary addiction to previously imprinted antigen. By contrast, the concomitant *de novo* antibody response is triggered by immune complex formation, capture, and presentation to B cells, both initially by subcapsular sinus macrophages (SSM) and then later by follicular dendritic cells (FDCs) during the subsequent germinal center (GC) reaction; and in all cases is catalyzed by innate immune cells. We suggest that this organization could serve in the design of vaccines tasked with offsetting primary antigen addiction/OAS where the antibody response can become locked into recalling pre-existing memory states that then dominate the composition of antibodies in circulation.

At the core of adaptive immunity, there is a balance between recalling past defenses (memory) and generating *de novo* (new) ones. Memory B cells are highly efficient at targeting pathogens they have previously encountered due to prior education and affinity maturation (Inoue, 2023; Inoue and Kurosaki, 2024; McHeyzer-Williams et al., 2000; McHeyzer-Williams and McHeyzer-Williams, 2005; Murugan et al., 2018). However, this advantage can become a limitation when facing rapidly hypervariable pathogens (such as influenza and SARS-CoV-2 viruses) when memory recall can become mismatched and new *de novo* responses from the germline (antigen naïve) B cell repertoire become increasingly important (Abreu et al., 2020; Dugan et al., 2020; Inoue, 2023; Inoue et al., 2021; Pape et al., 2021; Tennant et al., 2019). In this review, we discuss the factors that underscore this balance and present a model for its regulation by the innate immune system (Figure 1).

## Original antigenic sin (OAS) as a competitor to antigen-specific B lymphocytes within the primary B cell repertoire

Affinity-driven selection within B cell germinal centers (GCs) enhances the antigen binding strength of the B cell receptor (BCR), the membrane anchored precursor configuration of all antibodies (Chan and Brink, 2012; Ziegner et al., 1994). The process begins with initial complementarity between incoming antigen and the naïve BCR, where antigen binding strength is derived from primary diversification of the BCR clonal repertoire during B cell development and VDJ recombination (Berek and Milstein, 1987; Schatz and Swanson, 2011; Tonegawa, 1983). The antigen-specific B cell clones then seed the formation of (and are recruited to) GCs within the follicles of secondary lymphoid organs (such as lymph nodes or spleen) where affinity-driven selection occurs (Allen et al., 2007; De Silva and Klein, 2015; MacLennan, 1994; Victora and Nussenzweig, 2012; Victora and Nussenzweig, 2022). Key parameters regulating competitive fitness of individual GC B cell clones include their precursor frequency within the germline repertoire pool, their ‘starting’ germline affinity for cognate antigen and the available T cell help (Abbott and Crotty, 2020; Dosenovic et al., 2018; Gitlin et al., 2014; Schwickert et al., 2011; Victora and Nussenzweig, 2012; Victora and Nussenzweig, 2022). An important product of the GC reaction are the affinity matured memory B cells, defined as durable antigen-specific B cells that are recalled upon re-exposure to cognate antigen to rapidly differentiate into plasma cells that then generate the affinity matured antibodies (Dal Porto et al., 1998; Shinnakasu et al., 2016; Tas et al., 2016; Viant et al., 2020). Memory B cells persist within secondary lymphoid organs and circulate in the bloodstream, enabling them to mount a faster and robust response upon subsequent exposures to cognate antigen (Cancro and Tomayko, 2021; Dörner and Radbruch, 2005).

Despite the importance of B cell memory in immune defense, it also endows the phenomenon of OAS (Davenport et al., 1953; Pan, 2011; Virelizier et al., 1974a; Virelizier et al., 1974b). First identified by Thomas Francis Jr (Francis, 1960), OAS refers to the immune system’s tendency to recall immune responses against the primary antigen encountered, rather than generating a *de novo* affinity matured response after downstream exposure to variants of that primary antigen. OAS has often been described in the context of imprinting by influenza viral infection or vaccination, both in human and preclinical animal models, where collectively the work has emphasized the notion of ‘first flu is forever’ (Arevalo et al., 2020; Kim et al., 2012; Kim et al., 2009; Pan, 2011; Viboud and Epstein, 2016). Also referred to as ‘primary addiction’ (Schiepers et al., 2023), this feature can complicate the implementation of countermeasures against hypervariable pathogens, including influenza viruses and coronaviruses, that continue to evolve each season and ‘resist’ conventional vaccine design efforts (Amitai et al., 2020; Andrews et al., 2015; Cohen et al., 2022; Ellebedy and Ahmed, 2012; Gostic et al., 2016; Kaku et al., 2022; Leach et al., 2019; Ray et al., 2024; Sangesland et al., 2019; Sangesland et al., 2022; Sangesland and Lingwood, 2021; Schiepers et al., 2023; Weber et al., 2023). In some contexts, memory recall against the imprinted viral strain is protective (Gostic et al., 2016; Le Sage et al., 2021; Li et al., 2012; Lv et al., 2025) but may also serve to outcompete the generation of new/more broadened antibodies that target new antigen features (Goodwin et al., 2024; Pape et al., 2011; Schiepers et al., 2023). Immunologically, swift recall of affinity-matured memory B cells may lower the antigen that is available to the naïve B cell repertoire, effectively lowering the probability of affinity-maturing new B cell lineages with more diverse epitope specificities (Andrews et al., 2015; Pape et al., 2011; Schiepers et al., 2023; Wong et al., 2020). In addition to the competition posed by memory B cell recall, the associated serum antibody feedback also significantly modulates GC recruitment of naïve B cells from the repertoire (Cumpelik et al., 2021; Cyster and Wilson, 2024; Tas et al., 2022; Zhang et al., 2013). Here, circulating antibodies from primary responses regulate the GC participation of naïve B cells following secondary challenge, an effect that promotes or restricts GC recruitment depending on the antibody concentration, affinity, and epitope specificity (Forsell et al., 2017; Schaefer-Babajew et al., 2023; Zhang et al., 2016; Tas et al., 2022). These data point to memory recall (and its subsequent contribution to antibodies in circulation) as a regulator for (and sometimes competitor of) accessing the naïve B cell repertoire upon (repeat) exposure to antigen. We will argue that activation of naïve B repertoire, and its associated exploration of new antigen space is, in contrast to memory recall, both reliant and specifically catalyzed by effectors of innate immunity.

## Innate immunity and basic mechanisms of training

Innate immunity provides an immediate frontline of defense and is characterized by ‘hardwired’ receptors that recognize conserved pathogen-associated molecular patterns (PAMPs) and danger-associated molecular patterns (DAMPs) (Akira et al., 2006; Amarante-Mendes et al., 2018; Medzhitov and Janeway, 2000). Such pattern recognition receptors (PRRs) include Toll-like receptors (TLRs), NOD-like receptors (NLRs), and RIG-I-like receptors (RLRs) (Akira et al., 2006), which can be triggered to initiate broadly protective non-specific proinflammatory immune defenses (Iwasaki and Medzhitov, 2015; Janeway and Medzhitov, 2002). Cells providing innate immunity are devoid of adaptive immune receptors with antigen-specific memory (Janeway et al., 2001; Krangel, 2009; Tonegawa, 1983); however, heightened responses during secondary infection can occur through immune training (Kwong and Ordovas-Montanes, 2024; Netea et al., 2011; Ordovas-Montanes et al., 2020). A classic example of training includes the priming of mouse macrophages by Bacillus Calmette-Guérin (BCG) immunization, resulting in enhanced protection against a subsequent challenge with *Candida albicans* (Netea et al., 2011; VAN’t WOUT et al., 1992). In this pathway, prolonged cytokine signaling following BCG inoculation (e.g., mediated by IFN-γ, IL-1β, and TNF-α) drives both peripheral and progenitor-level reprogramming (Bickett et al., 2020; Hilligan et al., 2023; Hoft et al., 2018), producing an ‘inflammatory memory’ that enhances antimicrobial protection upon secondary immune challenge (Bickett et al., 2020; Kaufmann et al., 2018; Kong et al., 1997; Kwong and Ordovas-Montanes, 2024; Nahrevanian et al., 2013; Xu et al., 2024). In addition to macrophages, immune training is now considered a broad feature, occurring for other innate immune cell subsets (e.g., monocytes, dendritic cells, and natural killer cells) (Quintin et al., 2012; Saeed et al., 2014; Hole et al., 2019; Kleinnijenhuis et al., 2012; Sun et al., 2009), non-immune cells (e.g., epithelial cells, stromal fibroblasts, endothelial cells, and neurons epithelial cells) (Kazer et al., 2024; Naik and Fuchs, 2022), and has even been speculated to operate within B cells and T cells of the adaptive immune system (Kwong and Ordovas-Montanes, 2024; Ordovas-Montanes et al., 2020).

At the molecular level, trained immunity is defined by two principal features: (1) epigenetic reprogramming of hematopoietic progenitor cells in the bone marrow, and (2) long-lasting transcriptional and metabolic adaptations, both of which confer enhanced responsiveness to secondary stimulation (Netea et al., 2020; Netea et al., 2011). These changes can be triggered by diverse stimuli that engage PRRs that initiate pro-inflammatory signaling cascades (Ordovas-Montanes et al., 2020; Takeuchi and Akira, 2010). β-glucan, a fungal PAMP, is one of the most extensively characterized initiators of immune training (Ajit et al., 2024; Garcia-Valtanen et al., 2017; Geller and Yan, 2020; Netea et al., 2011). This molecule signals via the Dectin-1–Syk–CARD9–NF-κB axis, resulting in chromatin remodeling and enrichment of activating histone modifications such as H3K4me3 and H3K27ac at promoters of pro-inflammatory genes (e.g., *Tnf*, *Il6*, *Il1b*) (Quintin et al., 2012; Saeed et al., 2014). These epigenetic changes are reinforced by a metabolic shift toward aerobic glycolysis and glutaminolysis, resulting in accumulation of tricarboxylic acid (TCA) intermediates such as fumarate, which inhibit histone demethylases (such as KDM5) and stabilize the trained transcriptional landscape (Arts et al., 2016; Bekkering et al., 2018; Cheng et al., 2014). Functionally, the trained phenotype is underscored by augmented cytokine production and increased transcriptional accessibility upon secondary challenge, with effects persisting for several weeks in the absence of antigen-specific stimuli (Blok et al., 2015; Kleinnijenhuis et al., 2012). Collectively, these observations provide a view of innate immunity in which transcriptional plasticity enables innate immune reactions to be more durable and more potent upon repeated immune challenge.

## Evidence that innate immune reactions specifically catalyze expansion of the primary B cell repertoire and the hypothesis that this support could be reinforced by training

Unlike high-affinity antigen engagement by affinity matured memory B cells, antigen binding to the BCRs of the primary B cell repertoire is low affinity (~10^–6^ M) and is derived from primary repertoire diversification during VDJ recombination (Batista and Neuberger, 1998; Dal Porto et al., 1998; Eisen, 2014; Shah et al., 2018; Shih et al., 2002; Soto et al., 2019; Viant et al., 2020). However, this low-affinity binding is specifically supported by natural antigen display and activation modalities that are a direct function of innate immune reactions. While there is no known direct connection between B cell activation and immune training, we take the view that if training programs are generally conserved across cell types (Caldwell and Li, 2024; Hole et al., 2019; Kazer et al., 2024; Kleinnijenhuis et al., 2012; Kwong and Ordovas-Montanes, 2024; Naik and Fuchs, 2022; Ordovas-Montanes et al., 2020; Quintin et al., 2012; Saeed et al., 2014; Sun et al., 2009), then trained support of naïve B cell activation programs will also be enhanced upon repeat antigen exposure.

Following the entry of an external antigen, existing antibodies in circulation provide low-affinity emulsifications or ‘immune complexes’ with the antigen, marking it for further processing by innate immune reactions that help coordinate and catalyze the triggering of repertoire B cells (Heesters et al., 2013; Kim et al., 2024; McShane and Malinova, 2022; Phan et al., 2007; Ross, 1986; Schifferli and Taylor, 1989; Theofilopoulos and Dixon, 1980). Once bound, the immune complex activates complement via high-avidity interactions between circulating C1q and the Fc regions of the immune complexed antibodies (Bindon et al., 1988; Carroll, 2004; Duncan and Winter, 1988; Theofilopoulos and Dixon, 1980). Resultant proteolytic cascade of the complement pathway opsonizes the immune complexed antigen, making it a ligand for complement receptors (CR1 and CR2) (Carroll, 2004; Gonzalez et al., 2011; Griffin, 1980; Heesters et al., 2013; Heesters et al., 2016; Ricklin et al., 2010; Sondermann et al., 2001). When draining to the lymph nodes, the opsonized antigens are captured and extracted from solution by complement receptor 3 (CR3) displayed by the subcapsular sinus macrophages (SSM) lining this organ (Carrasco and Batista, 2007; Cyster, 2010; Gray and Cyster, 2012; Junt et al., 2007; Louie and Liao, 2019; Phan et al., 2007). A parallel innate immune receptor pathway that catalyzes antigen recognition of the low-affinity repertoire B cells includes the mannose-binding lectin (MBL) system, which promotes uptake of glycosylated protein antigen (such as subunit vaccine antigen) into B cell follicles (Martin et al., 2020; Read et al., 2022).

Multivalent surface presentation of receptors and/or ligands produces molecular synapses that serve to artificially enhance K_D_ through avidity effects (Batista et al., 2001; Batista and Neuberger, 2000; Fleire et al., 2006; Kato et al., 2020; Lingwood et al., 2012; Weaver et al., 2016). For repertoire B cells immunological synapses, enhancing affinity is formed at two stages (Figure 1): (1) between the SSM and naïve B cell to initially trigger BCR signaling and (2) between antigens presented for extended periods on the surface of follicular dendritic cells (FDCs) and the now activated B cells that undergo affinity maturation within GCs (Carrasco and Batista, 2007; Fischer et al., 1996; Gonzalez et al., 2011; Good-Jacobson and Shlomchik, 2010; Gray and Cyster, 2012; Harwood and Batista, 2010; Heesters et al., 2013; Heesters et al., 2014; Heesters et al., 2016; Jacobson et al., 2009; Kovács et al., 2021; Mongini and Inman, 2001; Phan et al., 2007; Victora and Nussenzweig, 2012). Within the immunological synapses, avidity is also coupled to receptor clustering and grouping effects (Carrasco and Batista, 2007; Harwood and Batista, 2010; Liu et al., 2012; Pierce and Liu, 2010; Tolar et al., 2008). The result is a more potent B cell activation than would be expected by binding to the individual germline BCR antigen binding sites (Batista and Harwood, 2009; Carrasco and Batista, 2007; Lingwood et al., 2012; Pierce and Liu, 2010; Ronsard et al., 2023).

## B cell germinal center lifespan is likely supported by innate immunity

Innate immune reactions both directly and indirectly regulate the dynamics of GCs, the sites in which the naïve repertoire expanded into new memory and high-affinity antibodies (Allen et al., 2007; Carrasco and Batista, 2007; Victora and Nussenzweig, 2012; Victora and Nussenzweig, 2022). A core example is IL-21, produced by T_FH_ cells that have been primed/instructed by dendritic cell input and is essential for maintaining Bcl-6 expression and the associated proliferative activity of GC B cells (Basso and Dalla-Favera, 2010; Linterman et al., 2010; Nutt and Tarlinton, 2011; Zotos and Tarlinton, 2012). In its absence, GCs collapse prematurely and fail to support effective affinity maturation and naïve B cell activation (Kim et al., 2025; Kotlarz et al., 2013; Linterman et al., 2010; Petersone and Walker, 2024; Zotos et al., 2010). Innate immune cells, including dendritic cells, macrophages, and SSM, also contribute cytokines such as IL-6, IL-15, and IL-1β within follicles, which promote B cell survival, class switching, and can sustain GCs after short periods of exposure (Dent et al., 1997; MacLennan, 1994; Tangye and Ma, 2020). IL-6 has been shown to sustain Bcl-6 expression in both T_FH_ and GC B cells, thereby supporting prolonged GC reactions (Betzler et al., 2023; Choi et al., 2013; Eto et al., 2011). Trained immunity is known to enhance these cytokine networks (e.g., IL1β, IL-6, and IFN-γ) (Arts et al., 2018; Divangahi et al., 2021; Kaufmann et al., 2018), suggesting a foundation reinforcing these GC effects.

## Memory recall of higher affinity B cells is less reliant on support from the innate immune system

Upon re-exposure to the same or similar antigen (secondary immune response), affinity matured memory B cells are quickly recalled, supplying the circulation with antibodies bearing tight complementarity (Palm and Henry, 2019; Phan et al., 2006; Syeda et al., 2024). Unlike naïve B cells which, due to their low-affinity BCRs, require robust co-stimulation and innate immune support for activation, memory B cells operate with independence, accelerating their recall and transition from quiescence to near-immediate expansion and differentiation into antibody-secreting plasma cells that produce high titers of affinity matured class-switched antibodies (IgGs) (Syeda et al., 2024; Zabel et al., 2014). While activated memory B cells can re-enter the GC for further affinity maturation, GCs that form during the secondary response are mostly composed of naïve repertoire B cells, functionally segregating these activation pathways (Callahan et al., 2024; Mesin et al., 2020; Shlomchik, 2018; Shlomchik and Weisel, 2012; Tarlinton and Victora, 2017; Victora and Nussenzweig, 2012; Weisel et al., 2016). The receptor structure of class-switched IgG^+^-BCR of circulating and resident memory B cells also enables faster signal transduction as compared to the IgM BCR (Liu et al., 2010; Xu et al., 2014). Furthermore, memory B cells exhibit pre-existing transcriptional and epigenetic alterations that prime them for this rapid effector function. For instance, they maintain upregulated expression of key transcription factors such as BLIMP-1 (B lymphocyte-induced maturation protein-1) and IRF4 (interferon regulatory factor 4), which are critical for driving plasma cell differentiation (Klein et al., 2006; Minnich et al., 2016; Shao et al., 2024). These modifications enable recalled memory B cells to differentiate into antibody-secreting plasma cells efficiently, even in the absence of strong co-stimulatory signals. This high efficiency, while providing rapid humoral immunity to recurrent antigen, also reinforces the primary addiction/OAS, locking the immune system into preferential recall of pre-existing memory states that dominate the composition of antibodies in circulation.

## Immune cell training and humoral immunity following immune challenge or vaccination

Baseline innate immune cell training programs, which are not antigen specific, can proceed in the absence/depletion of adaptive immunity, including within RAG-/- mice (Bhargavi and Subbian, 2024; Bosticardo et al., 2021; Priem et al., 2020) or when secondary lymphoid organs such as the spleen are removed (Ferreira et al., 2022). Similarly, training as a non-specific enhancer of protection following diverse immune challenges has been widely reported (Benn et al., 2013; Garly et al., 2003; Ter Steeg et al., 2021), although there are also varied results. For example, in preclinical models, BCG priming provides non-specific protection against influenza virus but not SARS-CoV-2 (Kaufmann et al., 2022), and the latter finding has been reproduced clinically (Pittet et al., 2023). However, our hypothesis is that an immune training stimulus on innate cells such as macrophages and DCs could serve to increase B cell repertoire-based diversification of antibodies, which in the context of OAS could serve to offset imprinted memory recall within the secondary response. Indeed, a clinical study has found that BCG priming can enhance humoral immunity elicited by the seasonal influenza vaccine when pre-existing immunity to the vaccine antigen is present (Leentjens et al., 2015). While in line with our hypothesis, it remains to be determined whether this enhancement occurred via elevated expansion and diversification from the B cell repertoire.

## Experimental predictions: Rational vaccine design to selectively expand the B cell repertoire and avoid primary addiction/OAS

Rational vaccine design refers to the deliberate selection and combination of antigens and immune modulators to direct specific cellular and molecular immune pathways (De Gregorio and Rappuoli, 2014; Ebensen and Guzmán, 2008; Rueckert and Guzmán, 2012). We suggest that this principle could be applied to reconfigure the early immune environment to favor the activation of naïve B cells and accelerate their recruitment into GCs and lower the antigen that is available for primary addiction/OAS in the secondary antibody response (Figure 1).

Rational vaccines could include use of trained immunity inducers such as β-glucan and BCG-derived molecules (Ajit et al., 2024; Blok et al., 2015; Rosati et al., 2024; Sánchez-Ramón et al., 2018; Uthayakumar et al., 2018) to reprogram the positioning, longevity, and innate immune functions, akin to the classic example of BCG providing a heightened innate response to a non-related secondary infection (Bickett et al., 2020; Kaufmann et al., 2018; Kong et al., 1997; Netea et al., 2011; VAN’t WOUT et al., 1992). SSMs could be conditioned to increase antigen retention via upregulated CR2/CD21 expression or enhanced surface mobility, thereby extending the duration over which naive B cells have access to intact antigens before they are rapidly consumed by memory clones (Iliopoulou et al., 2024; Junt et al., 2007; Phan et al., 2007; Phan et al., 2009). A similar enhancement could apply to FDCs that provide the antigen display surface for the GC reaction (Heesters et al., 2014; Heesters et al., 2021; Martínez-Riaño et al., 2023) and supply the chemokine CXCL13 directing B cell movement to and within the GC (Carlsen et al., 2004; Finch et al., 2013; Heesters et al., 2014). To further strengthen this pathway, trained immunity activators such as high-mannose oligosaccharides and mannan polymers (Borriello et al., 2022; Covián et al., 2019; Netea et al., 2011; Read et al., 2022) could also be used to engage MBL and initiate the lectin pathway of complement activation, amplifying both MASP-mediated opsonization of antigen and delivery to FDCs (Martin et al., 2020; Møller-Kristensen et al., 2007; Read et al., 2022).

Despite the efficiency of recalling previously imprinted B cells, the naïve B cell pool will numerically dominate the GC reaction (Mesin et al., 2020; Shlomchik, 2018), meaning that extended GC lifespan should favor diversifying both the antibodies generated and the memory B cell pool itself. Accordingly, trained immunity inducers could enhance the release of cytokines that would promote this effect (e.g., IL-21, IL-6, TNF-α, IFN-γ). Such ‘adjuvanting’ may not need to be delivered with the vaccine concomitantly. For example, administering immune training agents in a priming phase (Li et al., 2023; Sánchez-Ramón et al., 2018) may allow innate immune reactions to reach their functional peak before the antigen is administered, potentiating a broader window for the expansion of naïve B cell clones.

## Pitfalls

Rational vaccine design must also account for detrimental immune training scenarios that may lower antigen presentation activities and the capacity to expand the primary B cell repertoire. This includes defining the proper dosing regimens: low-dose priming can often maximize training responses, whereas elevated dosing with the same reagent can lead to tolerance reactions that lower immune cell responsiveness (Benn et al., 2013; Foster et al., 2007; Lajqi et al., 2023; Netea et al., 2016; Saeed et al., 2014). Similarly, chronic training by a rationally deployed vaccine (repeated immunization) could be detrimental since some chronic training stimuli result in epigenetic scarring/suppression of proinflammatory genes and are to be avoided, such as in the case of helminth infection which repositions dendritic cells into a regulatory phenotype that lowers antigen responsiveness (Ameur et al., 2025; Babu et al., 2006; Jankovic et al., 2004; Maizels and McSorley, 2016). More generally, environmental factors that tolerize immune reactions, such as smoking or air pollution, may limit effectiveness of the appropriate training stimuli (Lambrecht and Hammad, 2012; Saaoud et al., 2023); as could immunosenescence, the immune dysfunction and decline that accumulates with age (Franceschi et al., 2000; Jiang et al., 2025; Romero-Rodríguez et al., 2025). At the other extreme, long-term activation of innate immune training pathways should not be performed since this can lead to chronic disease states that are often underscored by prolonged exposure to proinflammatory cytokines such as IL-6 and associated with senescence and even cancer pathogenesis (Ahmad et al., 2022; Bekkering et al., 2021; Hirano, 2021; Hu et al., 2022; Yousif et al., 2021). Lastly, human males show enhanced microbial responsiveness following BCG vaccination (Koeken et al., 2020), pointing to sex differences as an additional variable that may govern the efficacy of rationally deployed training stimuli.

## Summary

We have presented a view of the humoral immune response in which the contribution of innate cells is biased for expanding the primary B cell repertoire, where antigen is accommodated at low affinity. This contrasts with a more limited role in their support for expanding higher affinity B cell memory, where the input will be restricted to the innate immune receptors expressed by the memory B cells themselves. Consequently, we have hypothesized that trained immunity is positioned to better service the early stages of B cell activation and development. This separation of innate immune reaction ‘cofactor’ function could serve in the design of vaccines tasked with offsetting primary antigen addiction and OAS.
